# Ultrasound Assessment of Postprocedural Arterial Pseudoaneurysms: Techniques, Clinical Implications, and Emergency Department Integration

**DOI:** 10.7759/cureus.43527

**Published:** 2023-08-15

**Authors:** Caitlin Azzo, Lachlan Driver, Katharine T Clark, Hamid Shokoohi

**Affiliations:** 1 Emergency Medicine, Harvard Medical School, Boston, USA; 2 Emergency Medicine, Massachusetts General Hospital, Harvard Medical School, Boston, USA

**Keywords:** trans-arterial access complications, yin-yang sign, femoral artery, temporal artery, point-of-care ultrasound, arterial pseudoaneurysms

## Abstract

This narrative aims to evaluate the efficacy of point-of-care ultrasound (POCUS) in the early diagnosis and management of postprocedural arterial pseudoaneurysms in the emergency department (ED). We hypothesize that POCUS can be used as the first line of imaging to distinguish vascular from non-vascular causes and diagnose a pseudoaneurysm if present. A comprehensive review of cases involving postprocedural pseudoaneurysms was conducted. We focus on patients who underwent endovascular procedures, including transfemoral and transradial arterial access for cardiac interventions, or received laceration repair after blunt head trauma. We analyzed each case's clinical symptoms, POCUS findings, and subsequent management. POCUS demonstrated high efficacy in early diagnosis by detecting pseudoaneurysm sacs with characteristic bi-directional flows (yin-yang sign) and, in some cases, partial thrombosis. The early identification of potential arterial complications allowed for efficient planning of further imaging and expedited surgical consultation, leading to timely and definitive management. Our study emphasizes the significance of using POCUS as the primary imaging modality for early detection and diagnosis of postprocedural arterial pseudoaneurysms. Incorporating POCUS into the initial assessment of patients presenting with pain and swelling at the site of arterial access or laceration repair can streamline consultation and potentially reduce the need for additional imaging, optimizing patient care in the ED setting.

## Introduction

The growth of arterial endovascular access is specifically focused on interventions targeting cardiac and neurovascular diagnosis, cardiac support, and extracorporeal circulation. This procedure involves the insertion of sterile catheters into blood vessels, notably the femoral or radial arteries. The shift towards these minimally invasive endovascular interventions for therapeutic purposes has subsequently increased access site complications. Post-intervention complications can range from site discomfort and hematomas to more severe issues such as the formation of pseudoaneurysms, arteriovenous fistulas, and dissections.

Pseudoaneurysm is a well-known iatrogenic arterial complication that can be accurately detected using point-of-care ultrasound (POCUS). Although radial and femoral arterial access is frequently obtained in the emergency department (ED), few studies have described the role of POCUS in the ED evaluation of pseudoaneurysms. In this case series, we illustrate that POCUS can be used as a first-line imaging modality for the detection of pseudoaneurysms following arterial endovascular interventions.

Pseudoaneurysms tend to develop with direct arterial trauma and differ from true aneurysms in that they are contained by only one or two of the three arterial wall layers (intima, media, and adventitia), as opposed to all three in a true aneurysm. Iatrogenic injury due to arterial access in percutaneous procedures is the most frequent cause of femoral and radial artery pseudoaneurysms [1]. The incidence of radial and femoral artery pseudoaneurysms varies in the literature and may depend on the indication for the procedure; however, it is less common to develop a pseudoaneurysm in the radial artery compared to the femoral artery after endovascular procedures. The reported incidence of radial and femoral artery pseudoaneurysm formation is less than 1% and 0.3-3.8%, respectively [2-5]. It has also been noted that the pseudoaneurysm complication rate is likely to be higher for therapeutic rather than diagnostic cases [6].

With increasing rates of ultrasonography, some society guidelines state that the acceptable rate of pseudoaneurysms post-procedural arterial endovascular access should be under 0.2% [7]. In a large retrospective study, the use of ultrasound guidance during endovascular procedures was associated with a 95% reduction in the rate of pseudoaneurysm formation [8,9]. Risk factors contributing to the formation of pseudoaneurysms include using a larger catheter size, a history of arterial hypertension, older patients (age > 65) undergoing the procedure, and the use of anticoagulation or antifibrinolytic therapy. Pseudoaneurysms are uncommon but can have serious complications. These may include emboli, rupture, necrosis of overlying tissues, and compression of nearby venous structures [10]. Even rarer are case reports of life-threatening rupture of a pseudoaneurysm after femoral arterial catheterization [11], delayed pseudoaneurysm diagnosis ultimately diagnosed with bedside ultrasound [12], and infected pseudoaneurysms caused by monitoring catheters [13].

## Case presentation

Case 1: iatrogenic radial artery pseudoaneurysm

An 81-year-old female presented to the ED with arm pain approximately one week after right radial artery catheterization for a scheduled percutaneous cardiac procedure. Her medical history was notable for atrial fibrillation on warfarin, chronic kidney disease, and aortic stenosis status post valve replacement. The patient initially experienced mild pain at the catheterization site that had steadily improved in the days following the procedure. However, on the day of presentation, the patient had acute sharp pain and increased swelling to the right wrist approximately eight hours before arrival in the ED. She had an uncomplicated recovery until this point, with no fever, chest pain, or changes to the sensation of the hand. She reported compliance with all medications, including warfarin.

**Figure 1:**
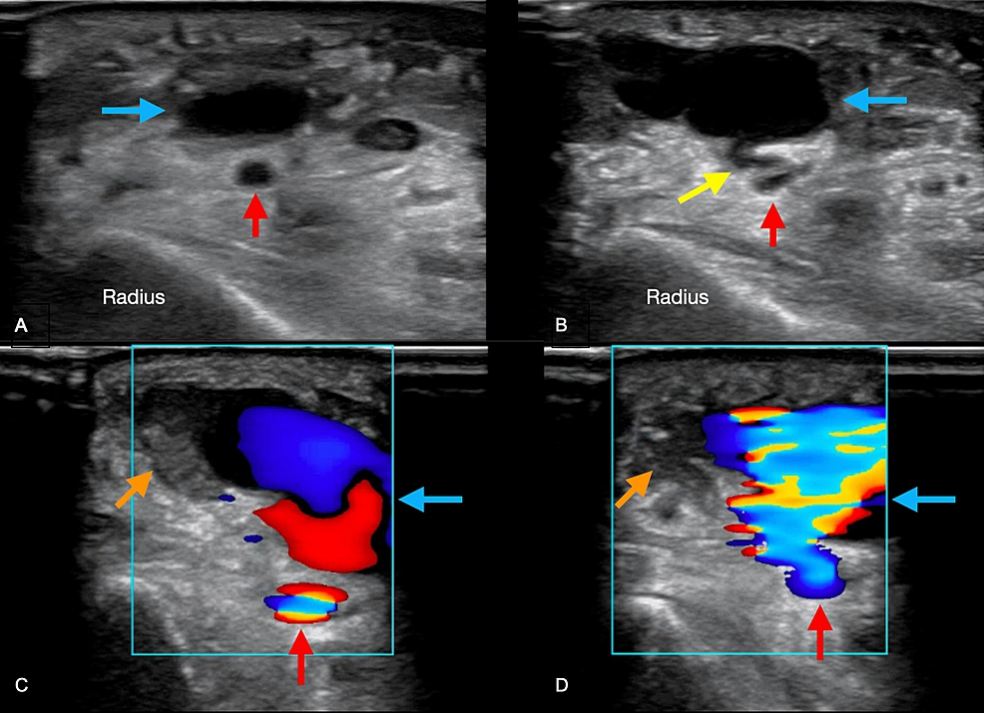
Pseudoaneurysm of the radial artery visualized by POCUS. (A) Linear probe in the transverse plane along the distal right forearm. The radial artery (red arrow) can be seen traversing the middle of the image with the distal radius below. (B) A large double-chambered pseudoaneurysm (blue arrow) connected to the radial artery below through an S-shaped pseudoaneurysm neck (yellow arrow). (C) The pseudoaneurysm is shown with positive color flow and a thrombus in the lateral chamber (orange arrow). A bidirectional flow pattern or “yin-yang” sign characteristic of pseudoaneurysms on ultrasound is visualized. (D) The pseudoaneurysm is shown in continuity with the right radial artery below.

Physical examination was notable for ecchymosis and pulsatile swelling, but there was no noticeable erythema or warmth along the volar aspect of the distal right forearm. Sensation was intact, but the patient could not fully range her wrist due to pain and swelling. Capillary refill in all five digits was less than one second. The emergency physicians performed a POCUS, revealing a pseudoaneurysm sac connected to the radial artery with a distinct neck (Figure 1). The Doppler flow examination revealed a sizeable two-chambered pseudoaneurysm with partial thrombosis in the outer chamber (Figure 1). No active extravasation was visualized. The vascular surgery team was immediately consulted, and the patient was admitted for operative repair of the radial artery pseudoaneurysm after a CT angiogram (CTA) confirmed the diagnosis. The patient responded well to surgery and was discharged after a short hospitalization.

Clinical Implication and Learning Points

1. Even standard procedures, like radial artery catheterization, can lead to complications such as pseudoaneurysms. Emergency medicine physicians should remain vigilant for delayed complications.

2. Patients on anticoagulation, such as warfarin, may be at higher risk for developing post-procedural pseudoaneurysms. This case emphasizes the importance of early detection of vascular complications in a patient on anticoagulation.

3. POCUS can quickly and non-invasively identify pseudoaneurysms. In this case, a rare two-chambered pseudoaneurysm with partial thrombosis is visualized by POCUS. In most cases, CTAs can give more details in preparation for surgical intervention.

Case 2: Iliofemoral arterial pseudoaneurysm following iliofemoral thrombectomy

A 75-year-old female with a history of peripheral vascular disease managed with two prior right inguinal cutdowns and thrombectomies presented with several days of progressively worsening right groin pain and bleeding. In the past year, the patient had two presentations for acute right limb ischemia requiring emergent right groin cutdown and thrombectomies of iliofemoral, profunda femoris, and superficial femoral arteries. She was discharged on aspirin and apixaban, with routine healing at follow-up appointments several weeks ago.

**Figure 2:**
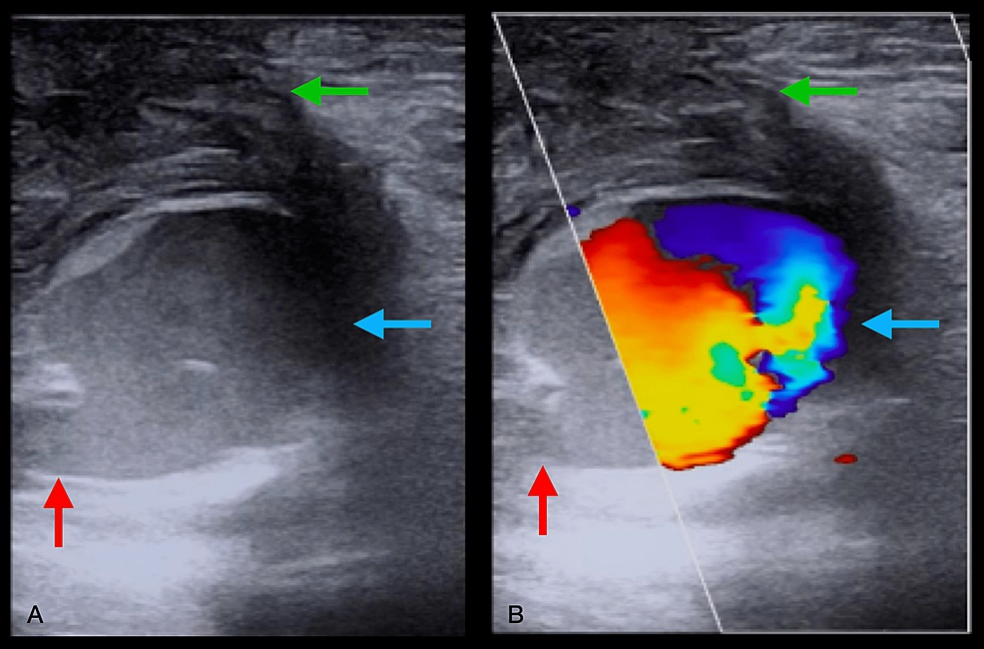
Pseudoaneurysm of right common femoral artery visualized by POCUS. (A) Linear probe in the transverse plane along the right inguinal area. The common femoral artery pseudoaneurysm (blue arrow) is seen in connection with the underlying artery (red arrow). Surrounding heterogenic material (green arrow) was later confirmed on CT to be infected hematoma. (B) Color Doppler imaging shows a “yin-yang” sign of the bidirectional flow pattern of pseudoaneurysms (blue arrow). The color Doppler box partially covered the cross-section of the pseudoaneurysm.

Her physical exam was notable for a small area of bluish discoloration over the right groin with mild bleeding just below the previous puncture site. Strength and sensation were intact in the bilateral lower extremities with 2+ distal pulses and good capillary refill bilaterally. A POCUS was performed with a linear transducer showing a right common femoral artery pseudoaneurysm measuring approximately 2.2 x 2.8 x 2 cm (Figure 2).

CTA imaging supported the diagnosis of right common femoral artery pseudoaneurysm measuring up to 4.6 cm with clotted blood and contrast filling the luminal component measuring up to 2.6 cm. In the OR, the patient was confirmed to have a right femoral artery pseudoaneurysm with an infected hematoma. The pseudoaneurysm was repaired, and she was started on antibiotics for her infection. Her treatment with apixaban, aspirin, and clopidogrel was resumed post-operatively. Her outpatient appointment with vascular surgery showed stable circulation.

Clinical Implication and Learning Points

1. Multiple interventions on a single vascular site can increase the risk of complications such as pseudoaneurysms. This patient's history of two thrombectomies, combined with anticoagulant use, likely predisposed her to develop a pseudoaneurysm.

2. While POCUS can provide initial evidence of a pseudoaneurysm, validating the findings using further imaging, such as CTA, may be necessary. In this case, a CTA has provided a more detailed visualization of pseudoaneurysm and defined a possible infected hematoma.

Case 3: superficial temporal artery pseudoaneurysm status post-laceration repair

**Figure 3:**
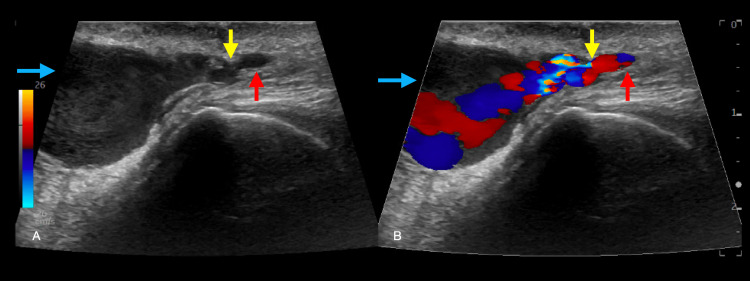
Pseudoaneurysm of the left superficial temporal artery. (A) Linear probe in the longitudinal plane along the left anterior temporal area, The left superficial temporal artery (red arrow), and adjacent pseudoaneurysm (blue arrow) that connected to the radial artery through a pseudoaneurysm neck (yellow arrow). (B) In the longitudinal view, the Doppler imaging of the left superficial temporal artery demonstrates a bi-directional color flow representing a yin-yang sign (blue arrow).

A 90-year-old male with a history of atrial fibrillation on apixaban, aortic stenosis, and hypertension presented to the ED with an expanding pulsatile mass and bleeding over the left forehead. Two weeks prior, the patient had sustained a laceration and extensive bleeding requiring repair with figure-of-eight sutures to achieve hemostasis. The head CTA at this time showed a forehead hematoma. He presented with spontaneous and atraumatic bleeding with a pulsatile mass at the site of prior injury, as well as tenderness over the site. A temporal artery ultrasound with a linear probe performed at the bedside by the ED provider in conjunction with the plastic surgery as well as a follow-up duplex study performed by radiology during the visit demonstrated bidirectional flow in a pulsatile mass. This was consistent with a pseudoaneurysm within the frontal branch of the left superficial temporal artery, measuring 1.9 x 1.4 x 2.7 cm (Figure 3). The patient was discharged one day later with an intact and hemostatic surgical site, without any complications post-temporal artery ligation.

Clinical Implication and Learning Points

1. Patients on anticoagulation, like apixaban in this case, may be at a higher risk for developing pseudoaneurysms after injuries.

2. An expanding pulsatile mass post-injury, especially in a vascular region like the forehead, should raise suspicion for a pseudoaneurysm.

3. While CTAs are valuable, they may not always detect pseudoaneurysms, especially if smaller or obscured by hematoma. A bedside ultrasound is an excellent tool for visualizing and confirming the presence of a pseudoaneurysm, evidenced by bidirectional flow in a pulsatile mass.

4. Pseudoaneurysms in the superficial temporal artery may be managed with ligation, as seen in this case, to prevent further complications.

Case 4: right femoral artery pseudoaneurysm four months after endovascular intervention

**Figure 4:**
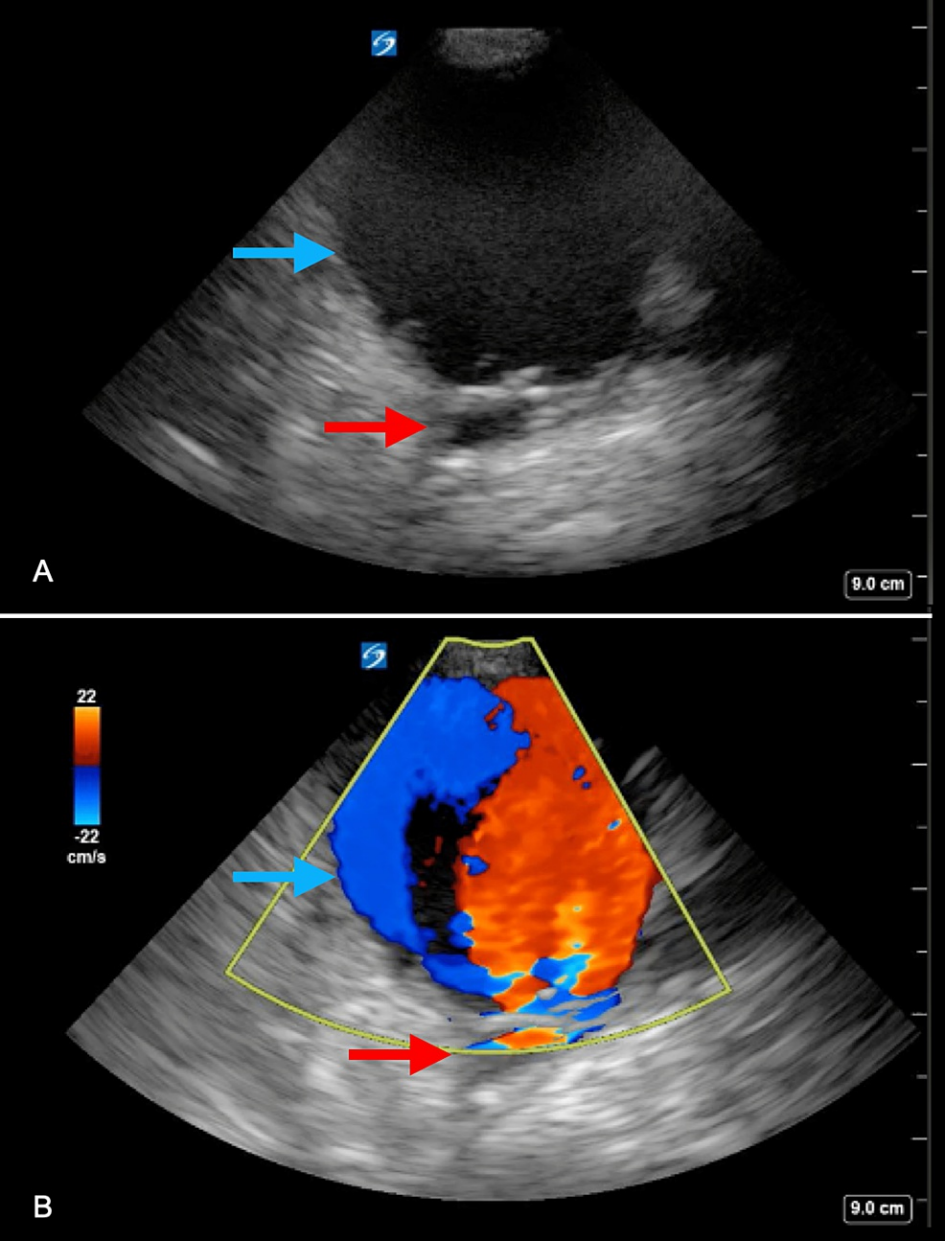
Large Pseudoaneurysm of the right common femoral artery. (A) Curvilinear probe in the transverse plane along the right inguinal area shows a common femoral artery pseudoaneurysm (blue arrow) with auto-contrast echogenicity from swirling flow in the pseudoaneurysm sac. (B) Color Doppler imaging illustrates the “yin-yang” flow pattern in this large pseudoaneurysm that overlies the common femoral artery (red arrow).

A 61-year-old female with a history of peripheral vascular disease and vasculitis with extensive surgical history on the arteries of her lower extremities presented to the ED with sharp pain in her right leg and groin. The patient had endovascular access on her right lower extremity four months prior. After the procedure, she experienced pain and discomfort in the right groin, which was initially diagnosed as a hematoma. On physical examination in the ED, she had a pulsatile mass to the right groin with faint pulses throughout the right lower extremity with no neurological deficits. A POCUS performed by the emergency physician revealed a large pseudoaneurysm with a sac measuring >6 cm, spontaneous auto-contrast, and a typical “yin-yang” appearance on color Doppler flow evaluation (Figure 4). Surgery was consulted, and the patient was taken for operative intervention.

Clinical Implication and Learning Points

1. The "yin-yang" sign in Doppler evaluation indicates pseudoaneurysms. Along with a large sac and auto-contrast, it leads to a rapid diagnosis. A curvilinear probe may be necessary for large pseudoaneurysms or specific body types.

2. A pseudoaneurysm >6 cm warrants urgent vascular consultation and likely intervention to prevent complications like rupture or thromboembolism.

Ultrasound Scanning Technique

POCUS is a valuable tool for assessing potential pseudoaneurysms in patients with pain and swelling at previous arterial access sites. A high-frequency linear transducer (6-12 MHz) is ideal, though a lower-frequency transducer (2-5 MHz) may be required for patients with edema or certain body habitus. Use an arterial or vascular preset for optimal frame rate and focus, adjusting the gain on B-mode.

When scanning the radial artery, progress from proximal to distal; for the femoral artery, move from distal to proximal. It is vital to ensure the transducer's pressure does not distort the pseudoaneurysm's diameter and avoid excessive pressure on a thin wall pseudoaneurysm.

A complete POCUS examination includes the evaluation of the focal or palpable abnormality concerning pseudoaneurysm. Once identified, slide the probe transversely from the adjacent artery toward the abnormality to visualize a pseudoaneurysm. Follow the artery and pseudoaneurysm transversely to view the connection and neck, and then rotate for a longitudinal view. If walls are unclear, use color Doppler for measurements. Measure the sac diameter using B-mode and color Doppler, and evaluate the pseudoaneurysm walls for flow defects and thrombosis.

In B-mode, pseudoaneurysms appear as anechoic fluid-filled structures with thin walls, distinct from the impacted artery, often showcasing swirling flows. They might exhibit thrombosis of varying echogenicity based on the thrombosis age. Record the dimensions of the pseudoaneurysm in anteroposterior, craniocaudal, and transverse orientations using the POCUS machine's caliper.

Apply color Doppler to observe pulsatility and connections, which is especially useful for detecting flow disturbances and potential AV fistulas. Pseudoaneurysms often exhibit a "yin-yang" bidirectional flow pattern. Partially thrombotic pseudoaneurysms often reveal blood flow through the thrombus, showing a false arterial lumen. Obtain spectral Doppler waveforms at a 45-60 degree angle, typically displaying a to-and-fro flow in the pseudoaneurysm neck.

## Discussion

Pseudoaneurysms, although relatively rare, with incidence generally reported under 4% in the literature [4,10,11], can result in significant morbidity [9-12] and must be considered in the differential diagnosis of a patient presenting with pain and swelling after an endovascular procedure or trauma. As demonstrated by this case series, POCUS in the ED may be considered after a physical examination to distinguish between vascular and non-vascular causes and may facilitate timely surgical consults and additional vascular imaging. Given that a post-operative hematoma, infection, or abscess may be an alternate etiology of painful swelling, POCUS can aid clinical decision-making to prevent the inadvertent incision and drainage of a pseudoaneurysm.

Though conventional angiography continues to be the gold standard in pseudoaneurysm diagnosis [14], POCUS is a safe and effective noninvasive modality for the diagnosis of pseudoaneurysms, with reported sensitivities and specificities of 94% and 94-97% from radiology literature [15], though more studies are needed to explore this imaging modality in the ED context. As demonstrated by this case series, POCUS in the ED can be an inexpensive, noninvasive, rapid, and safe modality to detect arterial pseudoaneurysms in conjunction with a physical examination.

## Conclusions

Our study underscores the significance of utilizing POCUS as the primary imaging modality for the early detection and diagnosis of postprocedural arterial pseudoaneurysms. In these four cases, POCUS demonstrated high efficacy in providing early diagnosis by detecting pseudoaneurysm sacs with characteristic bi-directional flows (yin-yang sign) and, in some cases, partial thrombosis. The early identification of potential arterial complications allowed for efficient planning of further imaging and expedited surgical consultation, leading to timely and definitive management. Although pseudoaneurysms are a relatively rare complication of arterial access and percutaneous interventions, they can result in significant morbidity. Thus, emergency physicians should be prepared to identify, diagnose, and initiate management of these severe complications in the ED. We believe that POCUS can be used as the first line of imaging to distinguish vascular from non-vascular etiologies and diagnose a pseudoaneurysm if present. In support of this, we demonstrated that the incorporation of POCUS can be used in early diagnosis of arterial pseudoaneurysm post arterial endovascular interventions or trauma.

Institution specific protocols for the incorporation of POCUS could include methods to identify the characteristic features of pseudoaneurysms, including an anechoic mass adjacent to the artery with swirling color flow and a bidirectional Doppler waveform in the pseudoaneurysm neck. With this knowledge, emergency physicians can effectively diagnose pseudoaneurysms in the ED. The incorporation of POCUS into the initial workup of such patients could streamline surgical consultation and the need for additional imaging. Future studies could evaluate whether POCUS can decrease the time to diagnosis, surgical evaluation, or the need for CT angiography in the ED. The cases presented above demonstrate that there could be a larger role for POCUS when it comes to this uncommon, though potentially severe, iatrogenic complication of obtaining arterial access for diagnostic or therapeutic purposes.
